# 5α-Hy­droxy­eudesm-4(15),11(13)-dien-8β,12-olide

**DOI:** 10.1107/S1600536812012470

**Published:** 2012-03-28

**Authors:** Xue Gao, Gang Chen

**Affiliations:** a Research Center of Medical Chemistry and Chemical Biology, Chongqing Technology and Business University, Chongqing 400067, People’s Republic of China

## Abstract

The title compound, C_15_H_20_O_3_, a sesquiterpene lactone, was isolated from the aerial parts of *Carpesium minus* Hemsl. (Compositae). The mol­ecule is composed of three rings, with the two cyclo­hexane rings in chair conformations and the cyclo­pentane ring adopting a twist conformation. The *A*/*B* ring junction is *trans*-fused. The absolute configuration shown has been arbitrarily assigned. In the crystal, mol­ecules are linked into [100] chains by O—H⋯O hydrogen bonds.

## Related literature

For the isolation and biological activity of the title compound, see: Lee *et al.* (2002 ▶); Yang *et al.* (2002 ▶); Li *et al.* (2011 ▶). For conformational analysis, see: Cremer & Pople (1975 ▶).
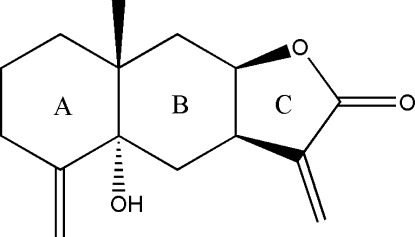


## Experimental

### 

#### Crystal data

C_15_H_20_O_3_
*M* *_r_* = 248.31
Monoclinic, 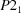

*a* = 7.893 (2) Å
*b* = 7.034 (2) Å
*c* = 12.166 (4) Å
β = 101.154 (3)°
*V* = 662.7 (3) Å^3^
*Z* = 2
Mo *K*α radiation
μ = 0.09 mm^−1^
*T* = 296 K
0.23 × 0.20 × 0.19 mm

#### Data collection

Bruker APEXII CCD diffractometer
Absorption correction: multi-scan (*SADABS*; Bruker, 2006 ▶) *T*_min_ = 0.981, *T*_max_ = 0.984
3673 measured reflections
1323 independent reflections
1159 reflections with *I* > 2σ(*I*)
*R*_int_ = 0.024

#### Refinement

*R*[*F* ^2^ > 2σ(*F* ^2^)] = 0.036
*wR*(*F* ^2^) = 0.084
*S* = 1.08
1323 reflections
165 parameters
1 restraint
H-atom parameters constrained
Δρ_max_ = 0.12 e Å^−3^
Δρ_min_ = −0.17 e Å^−3^

### 

Data collection: *APEX2* (Bruker, 2006 ▶); cell refinement: *SAINT* (Bruker, 2006 ▶); data reduction: *SAINT*; program(s) used to solve structure: *SHELXS97* (Sheldrick, 2008 ▶); program(s) used to refine structure: *SHELXL97* (Sheldrick, 2008 ▶); molecular graphics: *SHELXTL* (Sheldrick, 2008 ▶); software used to prepare material for publication: *SHELXTL*.

## Supplementary Material

Crystal structure: contains datablock(s) I, global. DOI: 10.1107/S1600536812012470/rn2102sup1.cif

Additional supplementary materials:  crystallographic information; 3D view; checkCIF report

## Figures and Tables

**Table 1:** Hydrogen-bond geometry (Å, °)

*D*—H⋯*A*	*D*—H	H⋯*A*	*D*⋯*A*	*D*—H⋯*A*
O1—H1*A*⋯O3^i^	0.82	2.06	2.868 (2)	168

Symmetry code: (i) 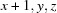.

**Fig. 1.**
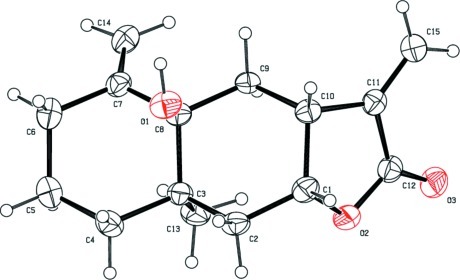
The molecular structure of the compound, with atom labels and 50% probability displacement ellipsoids.

## supplementary crystallographic information

### Comment

The title compound, 5α-hydroxyeudesm-4(15),11 (13)-dien-8β,12-olide (Fig.1), was isolated from the medicinal plant *Carpesium minus* (Compositae). This plant has been used to reduce swelling, relieve pain and as a detoxifying agent. The compound was identified by NMR spectra, which were compared with the previous reports (Lee *et al.*, 2002; Yang *et al.*, 2002; Li *et al.*, 2011). Herewith, we present its crystal structure.

The molecule of the title compound has three fused rings consisting of two six- and one five-membered rings (A/B/C). The A/B ring junction is *trans*-fused and B/C is *cis*-fused. The two cyclohexane rings have chair conformations with puckering parameters (Cremer & Pople,1975) Q = 0.571 (2) Å, θ = 175.7 (2)° and φ = 134 (4)° for the A ring and Q = 0.512 (2) Å, θ = 156.4 (2)° and φ = 344.9 (6)° for the B ring; the cyclopentane ring adopts a twist conformation with puckering parameters Q = 0.258 (2) Å and φ = 237.1 (4)°. In the crystal, the molecules are linked into chains by intermolecular O—H···O hydrogen bonds.

### Experimental

The air-dried whole plants of *Carpesium minus* (3.1 g) were pulverized and extracted with 95% EtOH and yielded 439 g of crude extract, which was then suspended in 2 *L* water. The suspension was partitioned with EtOAc (3×800 ml) to give a EtOAc-soluble portion, and a water-soluble fraction. After removal of the EtOAc under reduced pressure, 356 g of dark residue was obtained, and this was subjected to silica-gel chromatography, eluted with a stepwise gradient solvent system of petroleum/acetone 50: 1 to 0: 1 (*v*/*v*), to yield six major fractions (monitored by TLC). The third fraction (68 g) was rechromatographed on silica gel using a chloroform/MeOH (1: 0 to 30: 1) system and three fractions (Fr.A—Fr.C) were collected. Fr.B was further fractionated on a silica gel column using petroleum/EtOAc (3: 1) to give pure the title compound as colorless crystals.

### Refinement

All H atoms were placed in geometrically calculated positions, and allowed to ride on their parent atoms with O—H = 0.82 Å and C—H = 0.93–0.98 Å, and with *U*_iso_(H) = *xU*_eq_ (C), where *x* = 1.5 for methyl H atoms and hydroxyl group H atoms, and *x* = 1.2 for all other H atoms. In the absence of significant anomalous scattering, Friedel pairs were merged and the absolute configuration is arbitrary.

### Crystal data

**Table:**

C_15_H_20_O_3_	*F*(000) = 268
*M**_r_* = 248.31	*D*_x_ = 1.244 Mg m^−^^3^
Monoclinic, *P*2_1_	Mo *K*α radiation, λ = 0.71073 Å
*a* = 7.893 (2) Å	Cell parameters from 1583 reflections
*b* = 7.034 (2) Å	θ = 2.9–23.8°
*c* = 12.166 (4) Å	µ = 0.09 mm^−^^1^
β = 101.154 (3)°	*T* = 296 K
*V* = 662.7 (3) Å^3^	Block, colorless
*Z* = 2	0.23 × 0.20 × 0.19 mm

### Data collection

**Table:**

Bruker APEXII CCD diffractometer	1323 independent reflections
Radiation source: fine-focus sealed tube	1159 reflections with *I* > 2σ(*I*)
Graphite monochromator	*R*_int_ = 0.024
φ and ω scans	θ_max_ = 25.5°, θ_min_ = 2.9°
Absorption correction: multi-scan (*SADABS*; Bruker, 2006)	*h* = −9→9
*T*_min_ = 0.981, *T*_max_ = 0.984	*k* = −8→7
3673 measured reflections	*l* = −13→14

### Refinement

**Table:**

Refinement on *F*^2^	Primary atom site location: structure-invariant direct methods
Least-squares matrix: full	Secondary atom site location: difference Fourier map
*R*[*F*^2^ > 2σ(*F*^2^)] = 0.036	Hydrogen site location: inferred from neighbouring sites
*wR*(*F*^2^) = 0.084	H-atom parameters constrained
*S* = 1.08	*w* = 1/[σ^2^(*F*_o_^2^) + (0.046*P*)^2^ + 0.026*P*] where *P* = (*F*_o_^2^ + 2*F*_c_^2^)/3
1323 reflections	(Δ/σ)_max_ < 0.001
165 parameters	Δρ_max_ = 0.12 e Å^−^^3^
1 restraint	Δρ_min_ = −0.17 e Å^−^^3^

### Special details

**Table:**

Geometry. All e.s.d.'s (except the e.s.d. in the dihedral angle between two l.s. planes) are estimated using the full covariance matrix. The cell e.s.d.'s are taken into account individually in the estimation of e.s.d.'s in distances, angles and torsion angles; correlations between e.s.d.'s in cell parameters are only used when they are defined by crystal symmetry. An approximate (isotropic) treatment of cell e.s.d.'s is used for estimating e.s.d.'s involving l.s. planes.
Refinement. Refinement of *F*^2^ against ALL reflections. The weighted *R*-factor *wR* and goodness of fit *S* are based on *F*^2^, conventional *R*-factors *R* are based on *F*, with *F* set to zero for negative *F*^2^. The threshold expression of *F*^2^ > σ(*F*^2^) is used only for calculating *R*-factors(gt) *etc*. and is not relevant to the choice of reflections for refinement. *R*-factors based on *F*^2^ are statistically about twice as large as those based on *F*, and *R*- factors based on ALL data will be even larger.

### Fractional atomic coordinates and isotropic or equivalent isotropic displacement parameters (Å^2^)

**Table:**

	*x*	*y*	*z*	*U*_iso_*/*U*_eq_	
C1	0.5831 (3)	0.5040 (4)	0.2805 (2)	0.0451 (6)	
H1	0.5843	0.6432	0.2844	0.054*	
C2	0.6238 (3)	0.4471 (4)	0.1686 (2)	0.0462 (6)	
H2A	0.7088	0.5353	0.1504	0.055*	
H2B	0.5197	0.4618	0.1120	0.055*	
C3	0.6920 (3)	0.2446 (4)	0.16056 (19)	0.0404 (6)	
C4	0.7625 (4)	0.2265 (4)	0.0514 (2)	0.0534 (7)	
H4A	0.6677	0.2421	−0.0118	0.064*	
H4B	0.8442	0.3285	0.0486	0.064*	
C5	0.8506 (4)	0.0380 (5)	0.0400 (2)	0.0663 (9)	
H5A	0.8998	0.0396	−0.0272	0.080*	
H5B	0.7660	−0.0635	0.0325	0.080*	
C6	0.9931 (4)	−0.0002 (5)	0.1417 (2)	0.0619 (8)	
H6A	1.0384	−0.1275	0.1367	0.074*	
H6B	1.0868	0.0893	0.1425	0.074*	
C7	0.9243 (3)	0.0189 (4)	0.2486 (2)	0.0428 (6)	
C8	0.8428 (3)	0.2111 (3)	0.26178 (18)	0.0370 (5)	
C9	0.7793 (3)	0.2326 (3)	0.37155 (18)	0.0364 (5)	
H9A	0.8753	0.2109	0.4332	0.044*	
H9B	0.6931	0.1357	0.3752	0.044*	
C10	0.7006 (3)	0.4286 (3)	0.38653 (18)	0.0391 (5)	
H10	0.7921	0.5208	0.4142	0.047*	
C11	0.5786 (3)	0.4143 (3)	0.46605 (19)	0.0397 (5)	
C12	0.4025 (3)	0.4029 (3)	0.3975 (2)	0.0439 (6)	
C13	0.5451 (3)	0.0994 (4)	0.1587 (2)	0.0491 (6)	
H13A	0.4607	0.1147	0.0910	0.074*	
H13B	0.4917	0.1202	0.2222	0.074*	
H13C	0.5915	−0.0271	0.1618	0.074*	
C14	0.9291 (3)	−0.1238 (4)	0.3199 (2)	0.0514 (6)	
H14A	0.9759	−0.2401	0.3049	0.062*	
H14B	0.8857	−0.1079	0.3852	0.062*	
C15	0.6070 (3)	0.4053 (4)	0.5758 (2)	0.0516 (7)	
H15A	0.5149	0.3913	0.6127	0.062*	
H15B	0.7192	0.4128	0.6168	0.062*	
O1	0.9648 (2)	0.3595 (3)	0.25349 (15)	0.0503 (5)	
H1A	1.0515	0.3445	0.3021	0.075*	
O2	0.40859 (19)	0.4391 (3)	0.28997 (13)	0.0530 (5)	
O3	0.2681 (2)	0.3667 (3)	0.42676 (14)	0.0585 (5)	

### Atomic displacement parameters (Å^2^)

**Table:**

	*U*^11^	*U*^22^	*U*^33^	*U*^12^	*U*^13^	*U*^23^
C1	0.0446 (14)	0.0397 (13)	0.0508 (14)	0.0042 (11)	0.0082 (11)	0.0056 (12)
C2	0.0434 (13)	0.0513 (15)	0.0420 (13)	0.0031 (12)	0.0038 (10)	0.0127 (12)
C3	0.0365 (12)	0.0485 (15)	0.0358 (12)	0.0004 (11)	0.0058 (10)	0.0046 (11)
C4	0.0549 (15)	0.0681 (19)	0.0381 (13)	0.0003 (15)	0.0112 (11)	0.0055 (13)
C5	0.0695 (19)	0.083 (2)	0.0496 (16)	0.0125 (18)	0.0210 (14)	−0.0051 (15)
C6	0.0571 (17)	0.072 (2)	0.0608 (17)	0.0137 (15)	0.0213 (14)	−0.0028 (15)
C7	0.0332 (12)	0.0468 (14)	0.0473 (14)	0.0009 (11)	0.0050 (10)	−0.0027 (12)
C8	0.0299 (11)	0.0393 (12)	0.0405 (12)	−0.0064 (10)	0.0037 (9)	0.0032 (10)
C9	0.0331 (12)	0.0388 (13)	0.0346 (12)	−0.0009 (10)	−0.0001 (9)	0.0001 (10)
C10	0.0360 (12)	0.0373 (13)	0.0421 (12)	−0.0046 (11)	0.0030 (9)	−0.0010 (11)
C11	0.0380 (12)	0.0347 (12)	0.0463 (13)	−0.0018 (11)	0.0077 (10)	−0.0058 (10)
C12	0.0395 (13)	0.0425 (14)	0.0491 (14)	0.0017 (11)	0.0076 (10)	−0.0060 (11)
C13	0.0422 (14)	0.0570 (16)	0.0447 (14)	−0.0085 (12)	0.0003 (11)	−0.0044 (12)
C14	0.0465 (14)	0.0438 (15)	0.0634 (16)	0.0054 (12)	0.0096 (12)	−0.0006 (13)
C15	0.0537 (15)	0.0541 (16)	0.0471 (15)	0.0042 (13)	0.0099 (11)	−0.0043 (12)
O1	0.0357 (9)	0.0532 (11)	0.0608 (11)	−0.0118 (8)	0.0062 (7)	0.0067 (9)
O2	0.0357 (9)	0.0746 (13)	0.0470 (10)	0.0067 (9)	0.0036 (7)	0.0005 (9)
O3	0.0364 (9)	0.0772 (13)	0.0632 (11)	−0.0042 (9)	0.0130 (8)	−0.0094 (10)

### Geometric parameters (Å, º)

**Table:**

C1—O2	1.476 (3)	C7—C8	1.519 (3)
C1—C2	1.512 (3)	C8—O1	1.437 (3)
C1—C10	1.531 (3)	C8—C9	1.522 (3)
C1—H1	0.9800	C9—C10	1.538 (3)
C2—C3	1.533 (4)	C9—H9A	0.9700
C2—H2A	0.9700	C9—H9B	0.9700
C2—H2B	0.9700	C10—C11	1.495 (3)
C3—C4	1.541 (3)	C10—H10	0.9800
C3—C13	1.542 (3)	C11—C15	1.312 (3)
C3—C8	1.556 (3)	C11—C12	1.478 (3)
C4—C5	1.516 (4)	C12—O3	1.210 (3)
C4—H4A	0.9700	C12—O2	1.342 (3)
C4—H4B	0.9700	C13—H13A	0.9600
C5—C6	1.526 (4)	C13—H13B	0.9600
C5—H5A	0.9700	C13—H13C	0.9600
C5—H5B	0.9700	C14—H14A	0.9300
C6—C7	1.509 (3)	C14—H14B	0.9300
C6—H6A	0.9700	C15—H15A	0.9300
C6—H6B	0.9700	C15—H15B	0.9300
C7—C14	1.323 (4)	O1—H1A	0.8200
			
O2—C1—C2	110.7 (2)	O1—C8—C7	109.61 (18)
O2—C1—C10	104.44 (17)	O1—C8—C9	109.18 (19)
C2—C1—C10	117.9 (2)	C7—C8—C9	113.54 (19)
O2—C1—H1	107.8	O1—C8—C3	104.78 (17)
C2—C1—H1	107.8	C7—C8—C3	109.00 (19)
C10—C1—H1	107.8	C9—C8—C3	110.37 (17)
C1—C2—C3	116.2 (2)	C8—C9—C10	113.71 (18)
C1—C2—H2A	108.2	C8—C9—H9A	108.8
C3—C2—H2A	108.2	C10—C9—H9A	108.8
C1—C2—H2B	108.2	C8—C9—H9B	108.8
C3—C2—H2B	108.2	C10—C9—H9B	108.8
H2A—C2—H2B	107.4	H9A—C9—H9B	107.7
C2—C3—C4	108.73 (19)	C11—C10—C1	101.97 (17)
C2—C3—C13	110.1 (2)	C11—C10—C9	109.97 (19)
C4—C3—C13	109.2 (2)	C1—C10—C9	113.80 (19)
C2—C3—C8	108.32 (19)	C11—C10—H10	110.3
C4—C3—C8	108.73 (18)	C1—C10—H10	110.3
C13—C3—C8	111.67 (18)	C9—C10—H10	110.3
C5—C4—C3	113.5 (2)	C15—C11—C12	121.9 (2)
C5—C4—H4A	108.9	C15—C11—C10	131.1 (2)
C3—C4—H4A	108.9	C12—C11—C10	106.96 (19)
C5—C4—H4B	108.9	O3—C12—O2	121.7 (2)
C3—C4—H4B	108.9	O3—C12—C11	128.9 (2)
H4A—C4—H4B	107.7	O2—C12—C11	109.43 (19)
C4—C5—C6	111.1 (3)	C3—C13—H13A	109.5
C4—C5—H5A	109.4	C3—C13—H13B	109.5
C6—C5—H5A	109.4	H13A—C13—H13B	109.5
C4—C5—H5B	109.4	C3—C13—H13C	109.5
C6—C5—H5B	109.4	H13A—C13—H13C	109.5
H5A—C5—H5B	108.0	H13B—C13—H13C	109.5
C7—C6—C5	110.5 (2)	C7—C14—H14A	120.0
C7—C6—H6A	109.5	C7—C14—H14B	120.0
C5—C6—H6A	109.5	H14A—C14—H14B	120.0
C7—C6—H6B	109.5	C11—C15—H15A	120.0
C5—C6—H6B	109.5	C11—C15—H15B	120.0
H6A—C6—H6B	108.1	H15A—C15—H15B	120.0
C14—C7—C6	121.9 (2)	C8—O1—H1A	109.5
C14—C7—C8	124.4 (2)	C12—O2—C1	110.15 (17)
C6—C7—C8	113.6 (2)		
			
O2—C1—C2—C3	−83.6 (3)	C2—C3—C8—C9	60.6 (2)
C10—C1—C2—C3	36.5 (3)	C4—C3—C8—C9	178.6 (2)
C1—C2—C3—C4	−168.0 (2)	C13—C3—C8—C9	−60.9 (2)
C1—C2—C3—C13	72.4 (3)	O1—C8—C9—C10	56.0 (2)
C1—C2—C3—C8	−49.9 (3)	C7—C8—C9—C10	178.61 (18)
C2—C3—C4—C5	174.0 (2)	C3—C8—C9—C10	−58.7 (2)
C13—C3—C4—C5	−65.8 (3)	O2—C1—C10—C11	−26.1 (2)
C8—C3—C4—C5	56.2 (3)	C2—C1—C10—C11	−149.3 (2)
C3—C4—C5—C6	−54.6 (3)	O2—C1—C10—C9	92.3 (2)
C4—C5—C6—C7	52.6 (3)	C2—C1—C10—C9	−31.0 (3)
C5—C6—C7—C14	120.5 (3)	C8—C9—C10—C11	156.08 (18)
C5—C6—C7—C8	−56.6 (3)	C8—C9—C10—C1	42.4 (2)
C14—C7—C8—O1	127.5 (3)	C1—C10—C11—C15	−160.4 (3)
C6—C7—C8—O1	−55.5 (3)	C9—C10—C11—C15	78.5 (3)
C14—C7—C8—C9	5.1 (3)	C1—C10—C11—C12	22.0 (2)
C6—C7—C8—C9	−177.9 (2)	C9—C10—C11—C12	−99.1 (2)
C14—C7—C8—C3	−118.4 (3)	C15—C11—C12—O3	−7.9 (4)
C6—C7—C8—C3	58.6 (2)	C10—C11—C12—O3	170.0 (3)
C2—C3—C8—O1	−56.8 (2)	C15—C11—C12—O2	172.5 (2)
C4—C3—C8—O1	61.2 (2)	C10—C11—C12—O2	−9.7 (3)
C13—C3—C8—O1	−178.3 (2)	O3—C12—O2—C1	172.3 (2)
C2—C3—C8—C7	−174.07 (19)	C11—C12—O2—C1	−8.1 (3)
C4—C3—C8—C7	−56.1 (2)	C2—C1—O2—C12	149.8 (2)
C13—C3—C8—C7	64.5 (2)	C10—C1—O2—C12	22.0 (3)

### Hydrogen-bond geometry (Å, º)

**Table:**

*D*—H···*A*	*D*—H	H···*A*	*D*···*A*	*D*—H···*A*
O1—H1*A*···O3^i^	0.82	2.06	2.868 (2)	168

Symmetry code: (i) *x*+1, *y*, *z*.
